# Claudin-17 Deficiency Drives Vascular Permeability and Inflammation Causing Lung Injury

**DOI:** 10.3390/ijms26083612

**Published:** 2025-04-11

**Authors:** Mir S. Adil, Varun Parvathagiri, Abdulaziz H. Alanazi, Daulat Khulood, S. Priya Narayanan, Payaningal R. Somanath

**Affiliations:** 1 Program in Clinical and Experimental Therapeutics, College of Pharmacy, University of Georgia, Augusta, GA 30912, USA; 2 Research Division, Charlie Norwood VA Medical Center, Augusta, GA 30912, USA

**Keywords:** Cldn17, vascular permeability, inflammation, lung injury, pulmonary edema

## Abstract

The role of claudin-17 (Cldn17), a tight-junction protein, in vascular permeability remains unclear. We investigated the impact of Cldn17 suppression on vascular permeability. The Miles assay demonstrated significantly increased vascular permeability in the lungs and skin of *Cldn17^−/−^* mice, as evidenced by elevated Evan’s blue dye extravasation. The Matrigel plug assay demonstrated increased hemoglobin extravasation. Histopathological analysis revealed alveolar flooding, inflammatory cell infiltration, and lung injury in *Cldn17^−/−^* lungs. Wet/dry lung weight ratios indicated pulmonary edema, supporting the role of Cldn17 in pulmonary fluid balance, which was exacerbated with lipopolysaccharide administration. Ribosomal nucleic acid sequencing identified distinct transcriptional changes, with the principal component analysis showing clear clustering. Differential gene expression analysis highlighted significant alterations in inflammatory and metabolic pathways. Gene ontology and pathway enrichment analyses revealed the upregulation of immune-related processes, including leukocyte adhesion, interferon–gamma response, and neutrophil degranulation, alongside metabolic dysregulation affecting lipid transport and cytoskeletal organization. Reactome pathway analysis implicated Cldn17 in antigen presentation, interleukin-17 signaling, and inflammatory responses. These findings establish Cldn17 as a critical regulator of vascular permeability and immune homeostasis. Its deficiency drives vascular leakage, exacerbates lung injury, and alters immune signaling pathways, underscoring its potential as a therapeutic target for inflammatory lung diseases.

## 1. Introduction

Vascular permeability is a tightly regulated process that is essential for maintaining fluid balance, facilitating nutrient exchange, and supporting immune surveillance [1]. The endothelial and epithelial barriers, which form the structural interfaces of blood vessels and organ surfaces [2,3,4,5], play a crucial role in preventing excessive leakage while permitting selective molecular transport [6,7,8]. This regulation primarily depends on tight junctions (TJs), composed of transmembrane proteins such as claudins (Cldns). The claudin protein family includes at least 27 members, each with distinct tissue distributions and permeability characteristics [9,10]. While the roles of specific Cldns, such as Cldn5 in endothelial integrity and Cldn1 in epithelial function, are well-established, our previous study identified Cldn17 as one of the key Cldns modulated in the regulation of vascular permeability [11]. The physiological role of Cldn17 remains largely unexplored.

Cldn17 has been identified as a potential anion pore-forming protein, implicating its role in regulating anion balance across blood–tissue barriers [12]. We reported that Cldn17 contributes to electrolyte homeostasis and protection against oxidative stress, as evidenced by our findings from *Cldn17^−/−^* mice demonstrating kidney injury and increased reactive oxygen species, underscoring its role in renal physiology [13]. Given the importance of electrolyte balance and oxidative stress in maintaining vascular and epithelial integrity, these findings suggest that Cldn17 may play a broader function in regulating blood–tissue barrier function.

Disruptions in endothelial and epithelial barriers play a key role in the pathogenesis of inflammatory and vascular diseases, including acute lung injury (ALI) and pulmonary edema [14]. Barrier dysfunction facilitates dysregulated immune cell trafficking into tissues, driving chronic inflammation and tissue damage [14,15]. Under physiological conditions, endothelial cells tightly regulate immune cell extravasation, ensuring controlled migration in response to infection or injury. However, compromised TJ integrity increases permeability, allowing uncontrolled infiltration of leukocytes, cytokines, and inflammatory mediators, further amplifying inflammation and tissue injury [1,16]. Inflammatory signaling pathways play a pivotal role in endothelial dysfunction and TJ protein turnover [17]. Given the potential role of Cldn17 in maintaining junctional integrity and modulating inflammatory responses, elucidating its function in pulmonary pathophysiology is critically important.

While Cldns are increasingly recognized as key regulators of endothelial and epithelial barriers, the specific role of Cldn17 in preserving vascular integrity and immune homeostasis remains unclear. Understanding the molecular mechanisms by which Cldn17 contributes to the blood–tissue barrier’s function is crucial for identifying therapeutic targets for vascular leakage and inflammation-related diseases. By elucidating its function in endothelial and epithelial homeostasis, we seek to provide novel insights into its involvement in disease pathogenesis and explore potential therapeutic strategies.

## 2. Results

### 2.1. Cldn17 Is Expressed in Various Tissues, Albeit at Low Levels

**Figure 1:**
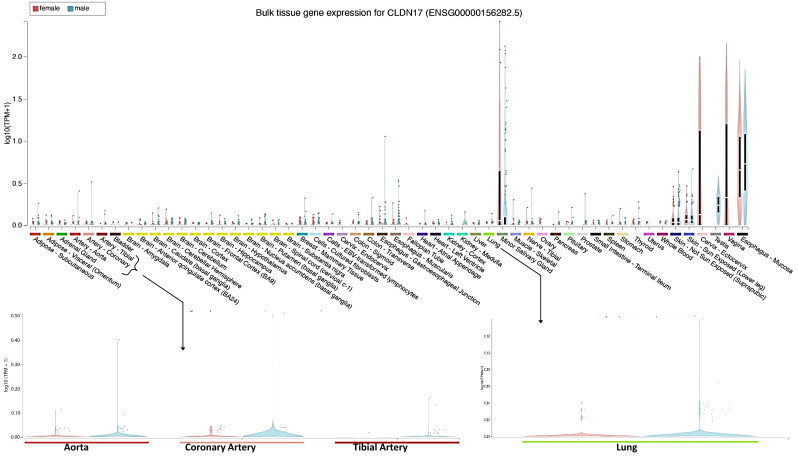
The Genotype–Tissue Expression (GTEx) portal analysis of Cldn17 in various human tissues shows predominant Cldn17 expression in tissues such as the salivary gland, esophagus, vagina, cervix, testes, and skin and modest expression in organs such as lungs, kidneys, and blood vessels.

To understand the tissue-wide distribution of Cldn17 in humans, we analyzed the Genotype–Tissue Expression (GTEx) portal (https://www.gtexportal.org/home/, accessed on 8 March 2025). The data revealed high expressions of Cldn17 in tissues such as the salivary gland, esophagus, vagina, cervix, testes, and skin (Figure 1), whereas while tissues such as the aorta, coronary artery, tibial artery, lung, kidney, and several other organs did express Cldn17, the expression levels were generally lower (Figure 1).

### 2.2. Reduced Cldn17 Expression in Lipopolysaccharide-Administered Mouse Lungs

**Figure 2:**
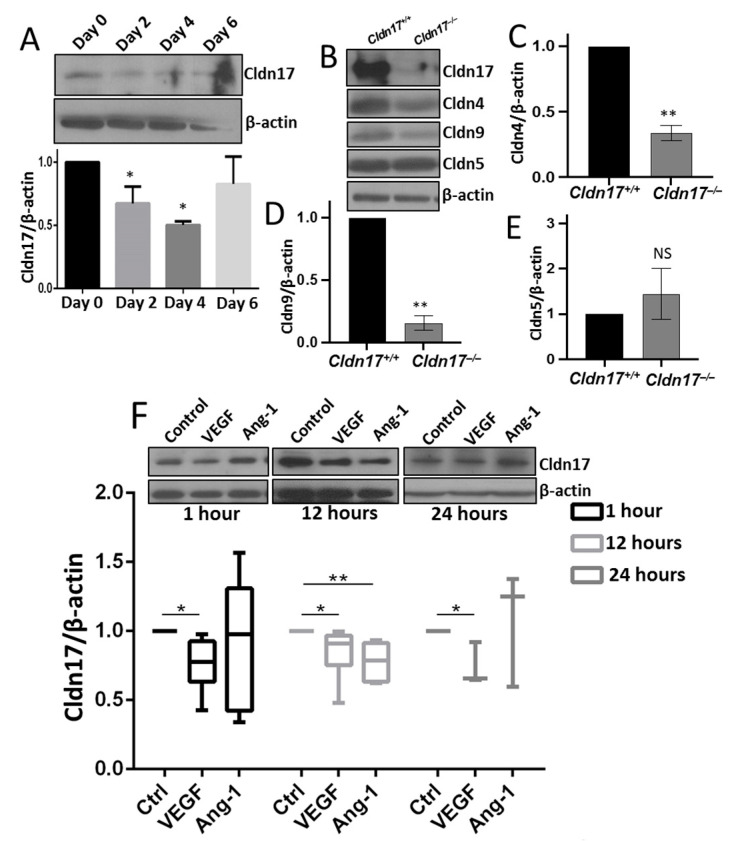
LPS-induced sepsis reduces Cldn17 expression in mouse lungs. (**A**) Representative Western blot images and band densitometry analysis showing reduced expression of Cldn17 in LPS-treated lungs on days 4 and 6 as compared with the vehicle-treated control (n = 3) (**B**–**E**) Representative Western blot images and quantification graph showing changes in the expression of Cldn4, Cldn5, and Cldn9 in the *Cldn17^−/−^* mouse lungs as compared with *Cldn17^+/+^* mouse lungs (n = 3). (**F**) Representative Western blot images and band densitometry analysis showing reduced expression of Cldn17 in lung endothelial cells treated with 20 ng/mL VEGF and 20 ng/mL angiopoietin-1 (Ang-1) at 1, 12, and 24 h after treatment (n = 3). Data are presented as mean ± SEM; NS, not significant; * *p* < 0.05; ** *p* < 0.01.

To assess Cldn17 protein expression in the lungs and examine the effects of lipopolysaccharide (LPS) administration, we performed Western blot analysis on mouse lungs administered with vehicle (sterile PBS) or 10 mg/kg LPS for 24 h (i.p.). Our results confirmed Cldn17 expression in the lungs and revealed its time-dependent modulation following LPS administration, with a reduction on days 2 and 4, followed by restoration on day 6 (Figure 2A). Additionally, the *Cldn17^−/−^* mouse lungs exhibited a significant reduction in Cldn4 and Cldn9 levels but showed no changes in Cldn5 (Figure 2B–E). In lung microvascular endothelial cells, VEGF treatment led to a modest yet significant reduction in Cldn17 expressions at 1, 12, and 24 h, whereas angiopoietin-1 treatment induced no significant changes (Figure 2F).

### 2.3. Cldn17 Deficiency Results in Vascular Permeability and Pulmonary Edema

**Figure 3:**
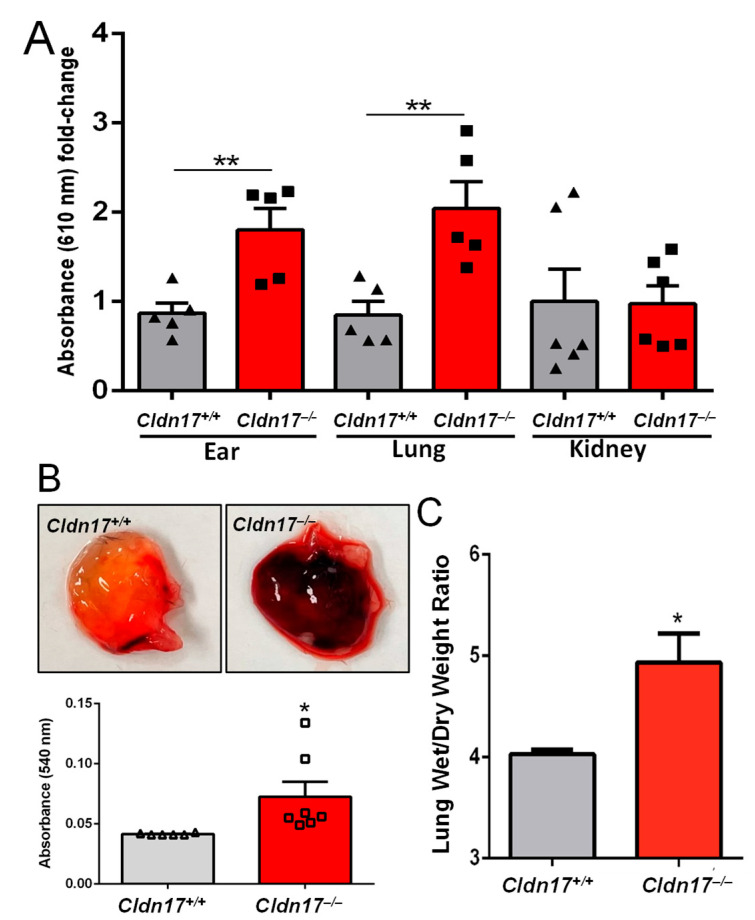
Matrigel plug and the Miles assay reveals hyperpermeability in *Cldn17^−/−^* mice. (**A**) Extravasation of Evan’s blue dye in various organs determined at a wavelength of 610 nm (n = 5–6). (**B**) Images of Matrigel plugs isolated from *Cldn17^+/+^* and *Cldn17^−/−^* mice and a bar graph showing absorbance of Matrigel plug lysates reflecting hemoglobin concentration (n = 6–7). (**C**) Pulmonary edema in *Cldn17^+/+^* and *Cldn17^−/−^* mice determined by wet/dry weight ratio (n = 6). Data are presented as mean ± SEM; * *p* ≤ 0.05; ** *p* < 0.01.

To assess vascular permeability, the Miles assay was performed on Cldn17^+/+^ and Cldn17^−/−^ mice via the intraperitoneal administration of Evan’s blue dye. Quantification of dye extravasation in the ears, lungs, and kidneys revealed a marked increase in vascular permeability in knockout animals, with significantly higher dye accumulation observed in the ears and lungs compared with the controls (Figure 3A). To further validate the role of Cldn17 in vascular leakiness, a Matrigel plug assay was performed. Hemoglobin concentration within subcutaneously injected Matrigel plugs was assessed using Drabkin’s reagent, with absorbance measured at 540 nm. Plugs isolated from the *Cldn17^−/−^* mice exhibited significantly elevated hemoglobin levels as compared with those from the *Cldn17^+/+^* counterparts, indicating increased vascular permeability (Figure 3B).

To evaluate lung pathology, age-, gender-, and weight-matched *Cldn17^+/+^* and *Cldn17^−/−^* mice were euthanized, and their lungs were collected. Pulmonary edema was assessed using a wet/dry ratio analysis, with lung weights measured before and after drying at 80 °C for 72 h. The *Cldn17^−/−^* groups showed significantly higher edema as compared with the *Cldn17^+/+^* controls, as demonstrated in Figure 3C.

### 2.4. Principal Component Analysis and Differential Gene Expression Identify Transcriptional Shifts and Dysregulated Genes in Cldn17^−/−^ Lungs

**Figure 4:**
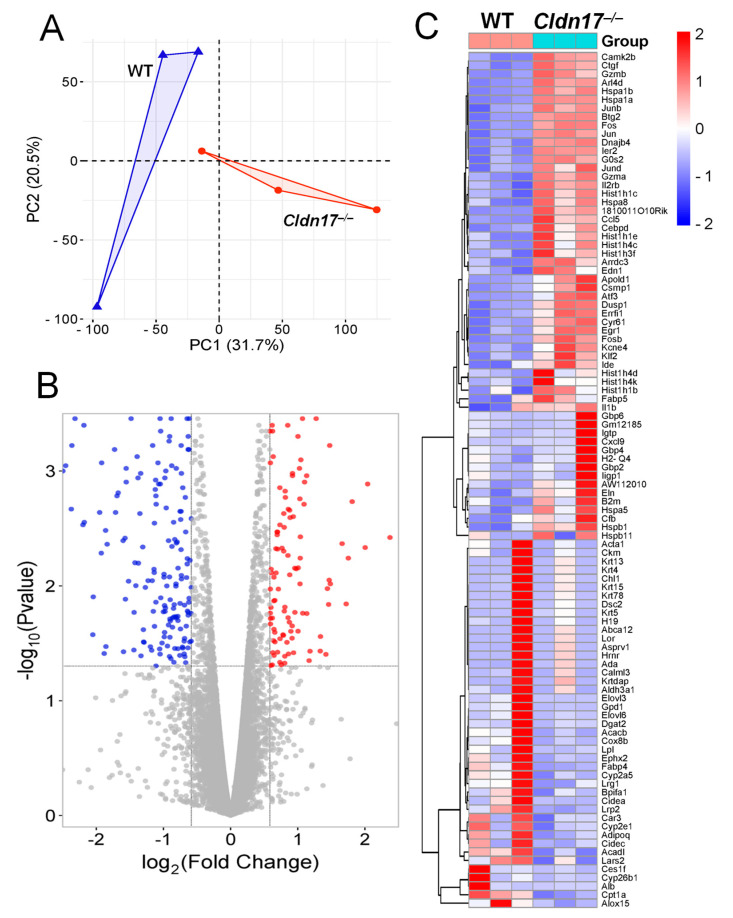
Analysis of DEGs in Cldn17 deficient samples. (**A**) Principal component analysis (PCA) displaying the fact that the two groups were not overlapped. (**B**) The volcano plot shows the upregulated and downregulated DEGs in the *Cldn17^−/−^* mice compared with the *Cldn17^+/+^* mice. Each dot represents a gene. (**C**) The heatmap demonstrates the top 100 DEGs among the two groups.

Principal component analysis (PCA) was performed to evaluate transcriptomic differences between the *Cldn17^+/+^* and *Cldn17^−/−^* samples (Figure 4A). The PCA plot reveals a clear separation between the *Cldn17^+/+^* and *Cldn17^−/−^* groups along PC1, which accounts for 31.7% of the variance. This separation suggests significant global transcriptional changes resulting from Cldn17 deletion. A volcano plot analysis was conducted to identify differentially expressed genes (DEGs) (Figure 4B). Genes that were significantly upregulated in the *Cldn17^−/−^* lungs are shown in red, while those that were downregulated are shown in blue. The distribution of DEGs highlights a distinct transcriptional shift, with numerous genes exhibiting significant expression changes upon Cldn17 deletion.

A heatmap of the top 100 DEGs (Figure 4C) further supports the substantial alterations in gene expression between the *Cldn17^+/+^* and *Cldn17^−/−^* groups. Clustering analysis reveals distinct expression profiles, with several genes exhibiting either upregulation or downregulation specifically in the knockout samples. Notably, genes involved in lipid metabolism and transport, such as *Abca12*, *Acacb*, and *Abcg2*, show significant differential expressions, suggesting that Cldn17 may play a role in regulating these pathways. The spreadsheet containing all the differentially regulated genes in *Cldn17^−/−^* compared to *Cldn17^+/+^* mouse lungs is included in the Supplemental Table S1.

### 2.5. Gene Ontology and Pathway Analysis Reveal Significant Enrichment in Vascular, Immune, and Metabolic Pathways in Cldn17^−/−^ Lungs

**Figure 5:**
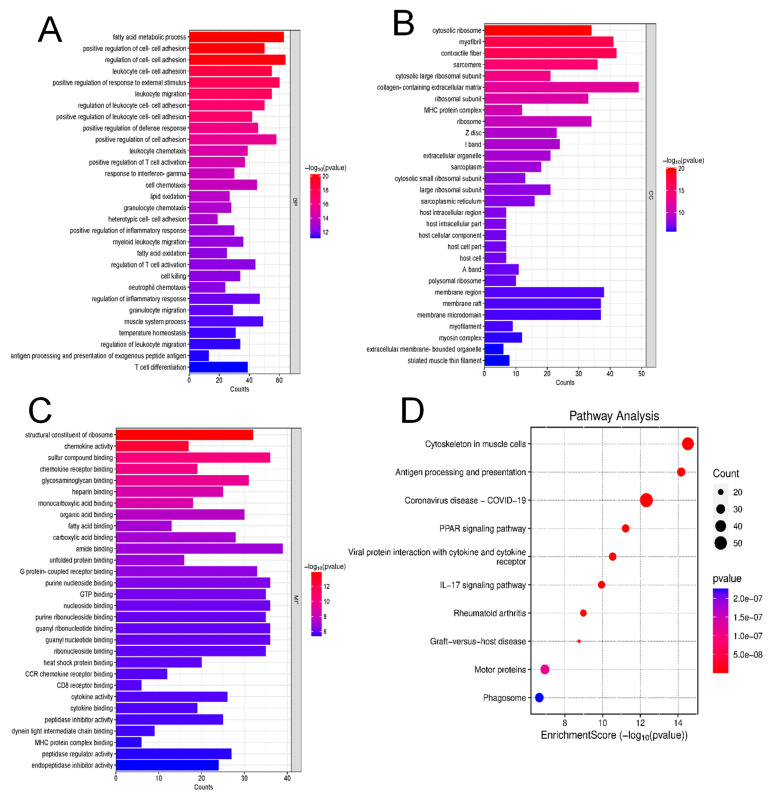
Gene ontology and pathway enrichment analysis revealed key biological processes and pathways affected by Cldn17 deficiency. (**A**–**C**) Gene Ontology (GO) analysis highlighting biological processes, cellular components, and molecular function significantly enriched in DEGs. (**D**) Pathway enrichment analysis using KEGG annotations, highlighting significantly affected pathways.

To investigate the biological pathways affected by our experimental conditions, all DEGs were subjected to complete Gene Ontology (GO) and pathway enrichment analyses. GO enrichment of biological processes (Figure 5A) revealed significant enrichment in immune-related pathways, including regulation of leukocyte cell–cell adhesion, myeloid leukocyte migration, and response to interferon–gamma. Additionally, pathways associated with fatty acid metabolism were also notably enriched.

GO analysis of cellular components (Figure 5B) highlighted enrichment in cytosolic ribosomes, extracellular matrix organization, and host cell components, suggesting alterations in cellular structure and interactions. Similarly, molecular function analysis (Figure 5C) identified significant enrichment in structural constituent proteins, receptor binding, and nucleotide binding functions, indicating changes in cellular communication and metabolic activity. KEGG pathway analysis (Figure 5D) confirmed key enrichment in antigen processing and presentation, IL-17 signaling, and pathways related to immune disorders such as rheumatoid arthritis and graft-versus-host disease. Notably, viral protein interactions with cytokine receptors and coronavirus-related pathways were also enriched, suggesting potential interactions with the host’s immune responses.

Furthermore, the Reactome pathway enrichment analysis (Supplemental Figure S1) demonstrated strong involvement of immune system pathways, including both innate and adaptive immune responses, as well as cellular responses to stimuli and lipid metabolism. Additional significant enrichment was observed in neutrophil degranulation, hemostasis, and signal transduction pathways. Similarly, the Wiki pathway enrichment analysis supports the significant dysregulations in signaling pathways associated with the immune system (Supplemental Figure S2). Together, these findings indicate that Cldn17 loss significantly impacts immune regulation, metabolic processes, and cellular signaling, which may drive inflammatory responses and disrupt cellular homeostasis.

### 2.6. Functional Enrichment Analysis Reveals Key Pathways Affected by Cldn17 Deletion

**Figure 6:**
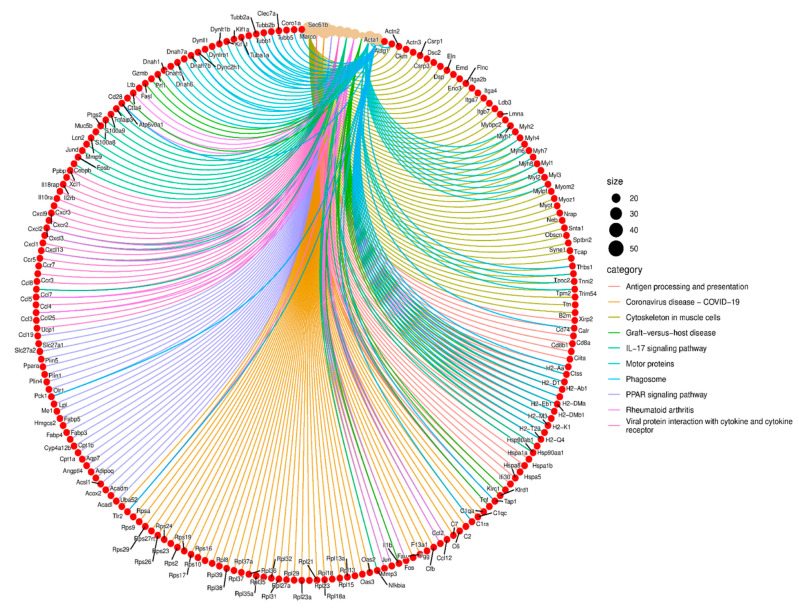
Circular chord diagram visualizing the functional enrichment of DEGs in the *Cldn17^−/−^* mouse lungs.

A circular chord diagram was generated to visualize the functional enrichment of DEGs (Figure 6). This analysis highlights several biological pathways significantly associated with the gene expression changes observed in the *Cldn17^−/−^* lungs. Each pathway is represented by a unique color, with connections illustrating the involvement of specific genes within these pathways.

Key enriched pathways include antigen processing and presentation, IL-17 signaling, phagosome formation, and viral protein interactions with cytokine receptors, indicating a strong immunological component to the transcriptional changes. Additionally, pathways related to cytoskeletal organization in muscle cells and motor proteins were affected, suggesting a potential role for Cldn17 in maintaining cytoskeletal integrity and facilitating cellular transport.

Several DEGs were involved in immune regulation, such as *H2-Ab1*, *Cd74*, *Cxcl9*, and *Il1b*, linking Cldn17 deletion to altered inflammatory responses. Moreover, genes associated with PPAR signaling and rheumatoid arthritis, such as *Fabp4* and *Ccl2*, were also dysregulated, further supporting CLDN17’s role in immune homeostasis and metabolic pathways. Lastly, we evaluated the gene–gene interactions between the DEGs. We noticed complex networks between these genes, which required further analysis to elucidate these interactions (Supplemental Figure S3). These findings suggest that CLDN17 deletion leads to widespread transcriptional changes that impact immune signaling, cellular architecture, and metabolic processes, providing novel insights into its functional role.

### 2.7. Gene Set Enrichment Analysis (GSEA) Reveals Deregulation of Pathways Involved in Vascular and Inflammatory Modulation in Cldn17^−/−^ Compared with WT Mouse Lungs

**Figure 7:**
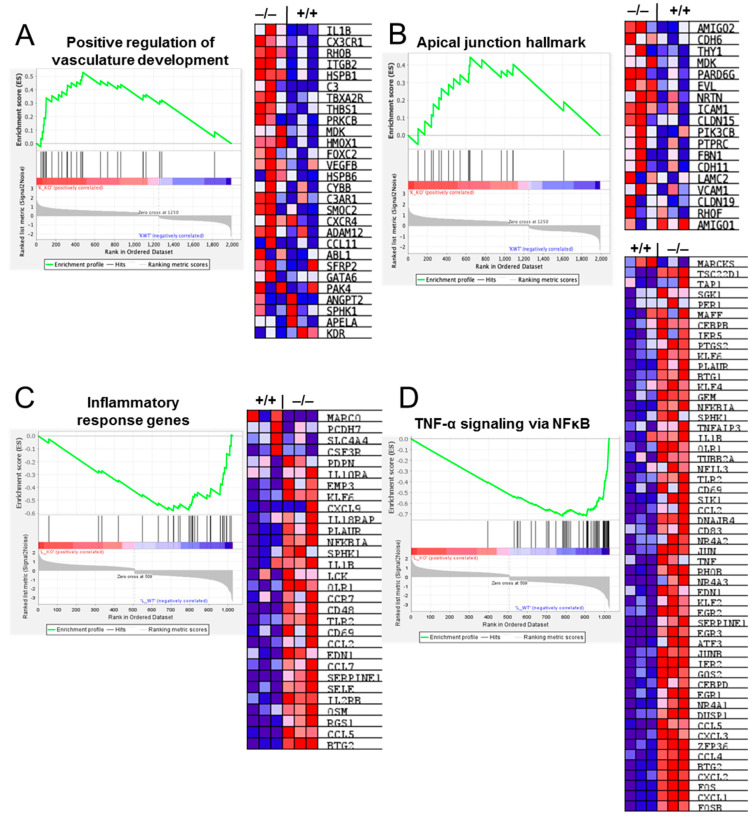
Heat maps and gene set enrichment analysis (GSEA) for altered genes involved in the regulation of (**A**) vascular development, (**B**) apical junction, (**C**) inflammatory response, and (**D**) TNFα and NFκB signaling. Data represented for genes with foldchange >2; n = 3; +/+; WT; −/−, Cldn17 knockout; *p* < 0.05.

GSEA revealed the enrichment of genes regulating several vascular modulatory pathways, such as positive regulation of vascular development and apical junction hallmark genes, in the lungs of the *Cldn17^−/−^* mice as compared with *Cldn17^+/+^* mice, which may impact vascular health (Figure 7A,B). Similarly, the analysis identified modulation of inflammatory-related signaling pathways, including chemokine signaling, TNF-α, and NFκB pathways, in the *Cldn17^−/−^* mouse lungs as compared with the *Cldn17^+/+^* mouse lungs (Figure 7C,D). Overall, these findings highlight an overactive inflammatory and vascular response in the *Cldn17^−/−^* lungs.

### 2.8. Cldn17 Deficiency Did Not Affect the Expression of Other Cell–Cell Junction Proteins in Mouse Lungs

**Figure 8:**
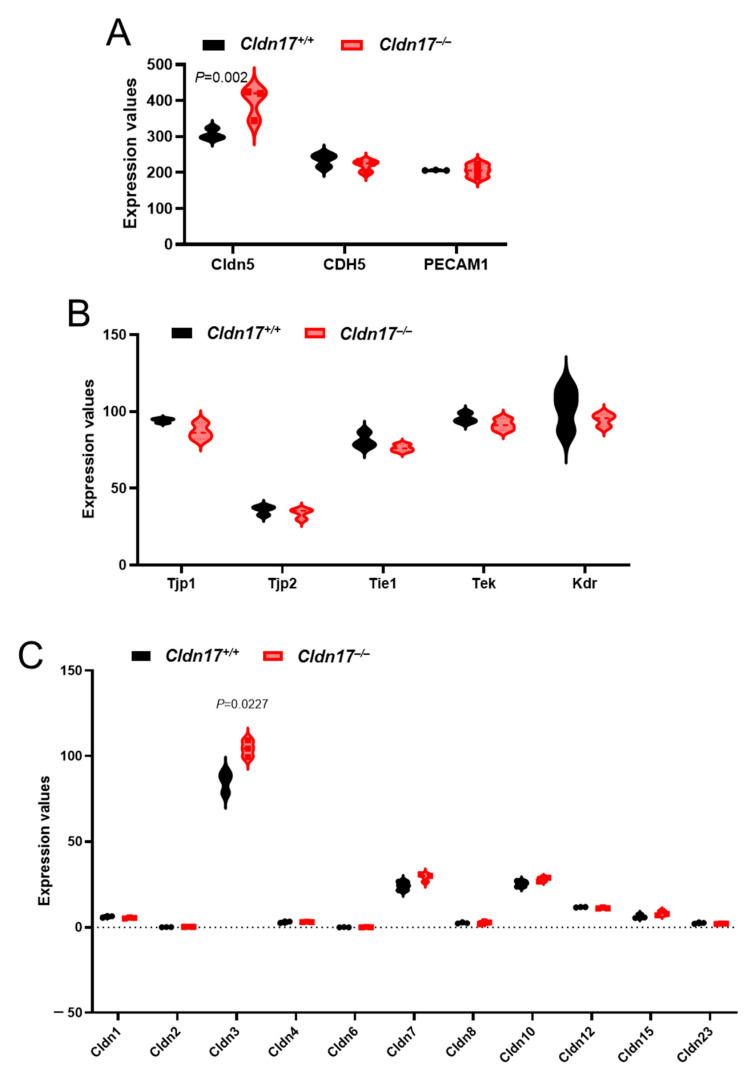
Cldn17 deficiency had no major impact on the expression of other cell–cell junction proteins in mouse lungs. (**A**) Violin plots showing changes in the expression of the major endothelial junctional and adhesion proteins Cldn5, Cdh2, and Pecam1 between the *Cldn17^+/+^* and *Cldn17^−/−^* mouse lungs. (**B**) Violin plots showing expression levels of the major endothelial markers Tjp1/Tjp2, Tie1, Tek, and Kdr between the *Cldn17^+/+^* and *Cldn17^−/−^* mouse lungs. (**C**) Violin plots showing no changes in the expression of other claudin isoforms in the *Cldn17^−/−^* mouse lungs as compared to the *Cldn17^+/+^* mouse lungs. n = 3; Data are presented as mean ± SEM.

To determine the potential impact of Cldn17 loss on the expression of other barrier-regulating proteins, we analyzed the RNA sequencing data for expression changes in the major endothelial cell junction proteins VE-Cadherin (Cdh5) as well as cell adhesion molecule Pecam1, revealing no changes in their expressions between the *Cldn17^−/−^* and *Cldn17^+/+^* mouse lungs except for a modest but significant increase in Cldn5 expression (Figure 8A). Similarly, no changes were observed in the expression levels of other endothelial markers such as zona occludens proteins (Tjp1 and Tjp2), Tie1, Tek, and Kdr (Figure 8B). Next, we analyzed changes in the expression levels of various claudins in the mouse lungs. Although we saw some expression changes in Cldn4 and Cldn9 in the Western blot analysis of the *Cldn17^−/−^* mouse lungs, the RNA sequencing data did not show any significant changes in the expression levels of any claudins in the *Cldn17^−/−^* mouse lungs as compared to the *Cldn17^+/+^* mouse lungs, except for a model increase in Cldn3, an isoform that is absent in endothelial cells (Figure 8C).

### 2.9. Cldn17 Deficiency Exacerbates LPS-Induced Lung Injury

The LPS-induced lung injury model was employed to investigate the impact of Cldn17 deficiency on lung injury, particularly given the immune-related activation observed in these mice during earlier transcriptomic analyses. By assessing baseline lung damage in the absence of LPS, we identified potential structural issues, prompting further exploration into how Cldn17 deficiency might exacerbate injury when exposed to LPS-induced inflammation. This approach allowed us to evaluate whether Cldn17 has a protective role in mitigating lung damage during inflammation.

**Figure 9:**
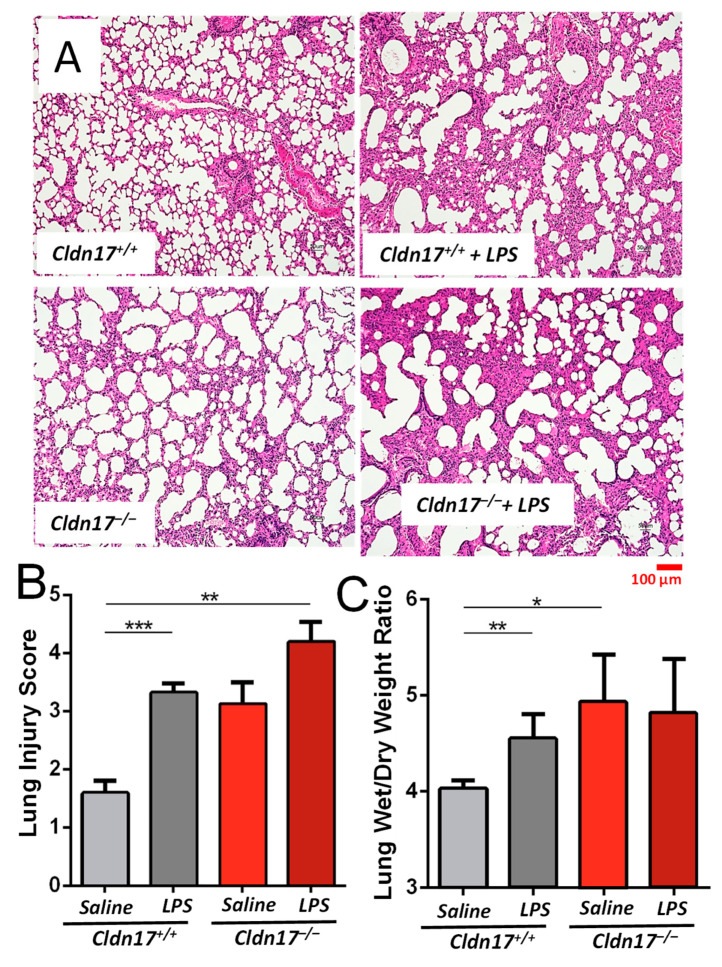
*Cldn17^−/−^* mouse lungs exhibit exacerbated LPS-induced ALI. (**A**) Representative H&E-stained lung sections from the *Cldn17^+/+^* and *Cldn17^−/−^* mice under saline and LPS-treated conditions. LPS treatment induced inflammatory cell infiltration and alveolar thickening. The *Cldn17^−/−^* lungs showed increased structural disruption and inflammation following LPS exposure. (**B**) Lung injury scores quantified from histological sections showed that LPS treatment significantly exacerbated lung injury in the *Cldn17^−/−^* mice compared with the *Cldn17^+/+^* mice. (**C**) Bar graph showing lung wet/dry weight ratio of the LPS-treated *Cldn17^−/−^* mice, which exhibited significantly higher ratios compared with the *Cldn17^+/+^* mice, indicating increased vascular permeability and fluid accumulation. * *p* < 0.05; ** *p* < 0.01; *** *p* < 0.001. n = 4 for *Cldn17^+/+^*; and n = 3 for *Cldn17^−/−^*. Data are presented as mean ± SEM.

Representative hematoxylin and eosin (H&E) stained lung sections (Figure 9A) show lung morphology across different experimental groups. Lungs from the *Cldn17^+/+^* mice appeared normal with minimal inflammation, whereas LPS treatment in WT mice induced increased cellular infiltration and thickened alveolar walls. Notably, the *Cldn17^−/−^* lungs displayed mild structural abnormalities even without LPS exposure, suggesting a potential baseline defect. After LPS treatment, the *Cldn17^−/−^* mice exhibited extensive inflammatory cell infiltration, alveolar wall thickening, and structural disorganization, indicating aggravated lung injury compared with the *Cldn17^+/+^* LPS-treated mice.

Quantification of lung injury (Figure 9B) revealed a significant increase in injury scores following LPS administration across all genotypes. Importantly, the *Cldn17^−/−^* groups with LPS exhibited significantly higher scores compared with WT with LPS, suggesting that Cldn17 deficiency heightens susceptibility to LPS-induced lung damage.

The lung wet-to-dry weight ratio, an indicator of pulmonary edema, was significantly elevated in the *Cldn17^−/−^* mice, especially following LPS administration (Figure 9C). These findings suggest increased vascular permeability and fluid accumulation in the *Cldn17^−/−^* mice, further supporting Cldn17’s role in maintaining the endothelial barrier’s integrity. Overall, these results indicate that Cldn17 plays a critical role in lung homeostasis and its deficiency exacerbates inflammation, lung injury, and vascular leakage in response to LPS challenge.

### 2.10. Cldn17 Deficiency Leads to Inflammation and Leukocytosis in Mice

**Figure 10:**
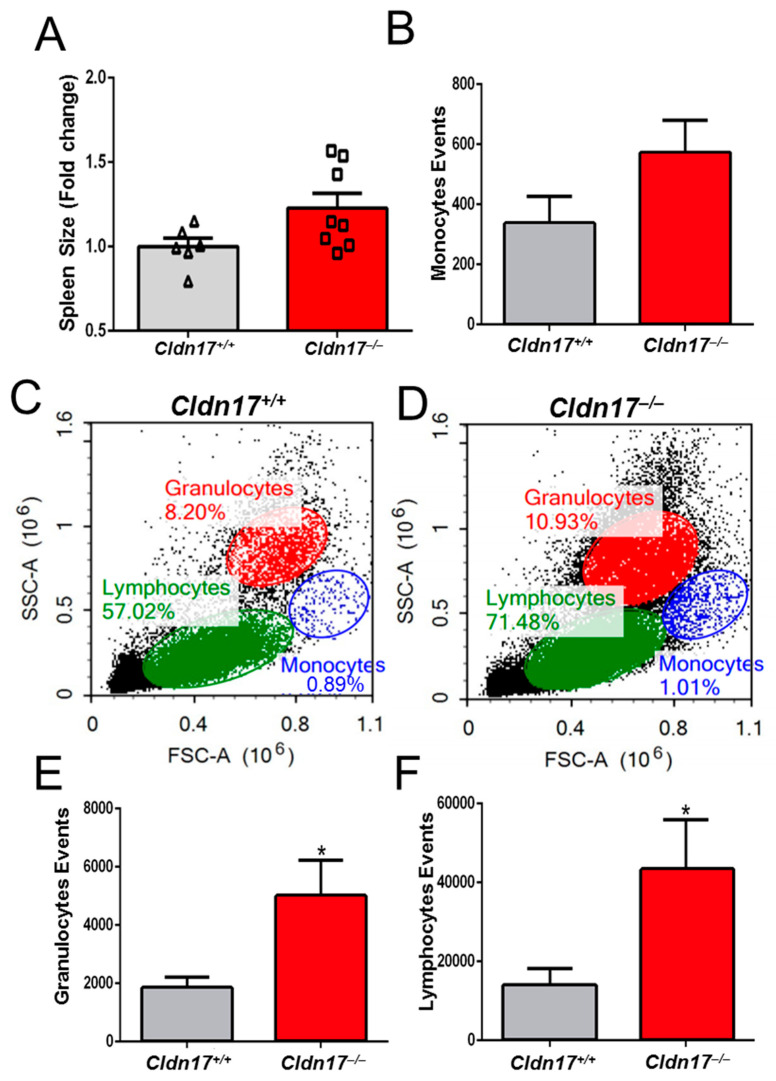
Cldn17 deficiency is linked to splenomegaly and increased expansion of granulocytes and lymphocytes. (**A**) Increased spleen size in the *Cldn17^−/−^* vs. *Cldn17^+/+^* mice (n = 6–8). (**B**) Monocytes events. (**C**,**D**) Forward vs. side scatter plots for the *Cldn17^−/−^* vs. *Cldn17^+/+^* groups. (**E**,**F**) Bar graphs showing granulocytes and lymphocyte events. Data are presented as mean ± SEM; WBC, white blood cells; * *p* < 0.05.

**Figure 11:**
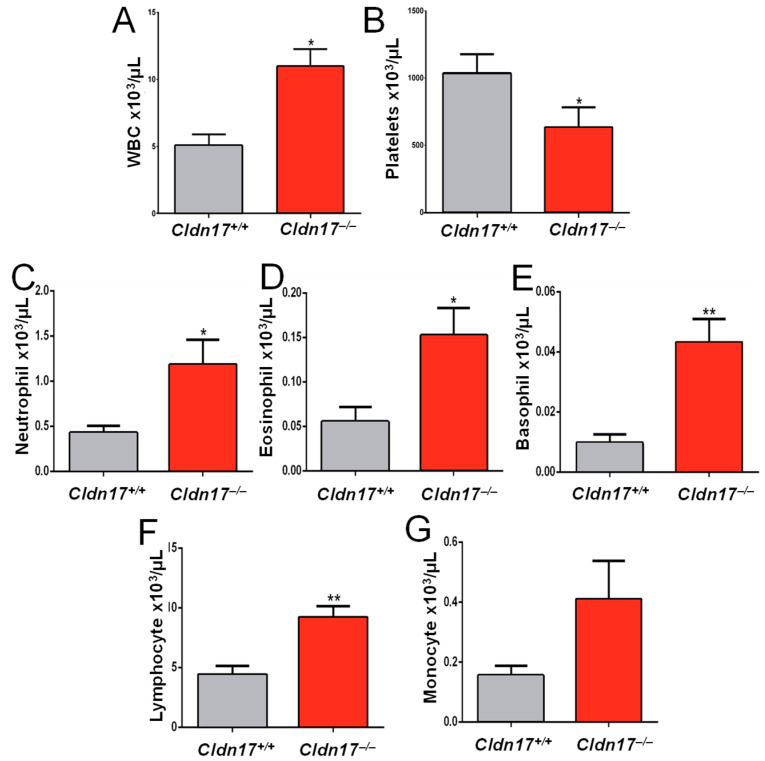
Loss of Cldn17 causes leukocytosis. Bar graphs showing (**A**) WBCs (n = 7), (**B**) differential count (n = 7), and (**C**) platelets (n = 8) in the *Cldn17^−/−^* vs. *Cldn17^+/+^* mice. (**D**–**G**) Bar graphs showing increased eosinophil, basophil, total lymphocytes and monocytes in *Cldn17^−/−^* vs. *Cldn17^+/+^* mouse blood, respectively (n=7). Data are presented as mean ± SEM; WBC, white blood cells; * *p* < 0.05; ** *p* < 0.01.

While spleen weight showed an increasing trend in the *Cldn17^−/−^* mice, the difference was not statistically significant (Figure 10A). Given the potential for an inflammatory response in the *Cldn17^−/−^* mice, as evidenced by the RNA sequencing data, we performed flow cytometry analysis on fresh tissue samples. Monocytes exhibited an increasing trend (Figure 10B), while granulocytes and lymphocytes were significantly elevated in the *Cldn17^−/−^* mice (Figure 10C–F), indicating leukocytosis in the knockout group. To validate these findings, we conducted a complete blood count (CBC) analysis using blood collected via retro-orbital puncture from both the WT and the *Cldn17^−/−^* animals. The results confirmed a more than twofold increase in WBCs and a reduction in platelet count (Figure 11A,B). Differential blood count further revealed significantly elevated neutrophils, lymphocytes, eosinophils, and basophils in the *Cldn17^−/−^* mice compared with the *Cldn17^+/+^* controls (Figure 11C–G). These findings demonstrate that Cldn17 deficiency induces leukocytosis and thrombocytopenia, highlighting its role in immune regulation and vascular homeostasis.

## 3. Discussion

Cldns are essential transmembrane proteins that form TJs, regulating paracellular permeability and maintaining barrier integrity in epithelial and endothelial tissues [10]. In vascular homeostasis, Cldns such as Cldn5 play a crucial role in controlling the endothelial barrier function and preventing excessive leakage of fluids and solutes [10,11]. Additionally, Cldns contribute to inflammation homeostasis by modulating immune cell infiltration and cytokine signaling, influencing both protective and pathological inflammatory responses [18,19]. Dysregulation of Cldns can lead to increased vascular permeability, exacerbating conditions like edema, organ injury, and other chronic inflammatory diseases [10,11,18,20,21,22,23,24,25]. Among the 27 known Cldns, only a few have been well-characterized for their specific roles in physiology and pathology. Cldn17, identified as a paracellular anion channel [26], remains largely uncharacterized in terms of its functional significance. Cldn17 expression is highly modulated in response to changes in vascular permeability [11]. This study provides compelling evidence for the critical role of Cldn17 in maintaining vascular integrity and regulating immune responses. Our findings demonstrate that Cldn17 deficiency in mice leads to increased vascular permeability, pulmonary injury, and exacerbated LPS-induced lung inflammation. Published studies and publicly available datasets indicate that Cldn17 expression levels are lower in most tissues, suggesting tight regulatory control and/or a potential rate-limiting role. Furthermore, we observed significant transcriptional changes and immune pathway dysregulation in the *Cldn17^−/−^* mouse lungs, highlighting the multifaceted impact of Cldn17 on cellular homeostasis.

While the majority of Cldns, such as Cldn5, serve primarily structural roles in modulating the paracellular barrier, fewer Cldns function as ion channels [10]. Among the paracellular ion channel-forming Cldns, anion-selective channels are limited to three isoforms: Cldn10b [27], Cldn17 [26], and Cldn4 [28]. Unlike structural claudins, which provide strength and are expressed at higher levels, paracellular ion pore-forming claudins, such as Cldn17, are expressed in lower quantities [10]. Our previous analysis of endothelial-cell claudin expression identified Cldn17 in endothelial cells, whereas Cldn10 and Cldn4 were undetected [11], suggesting a crucial role for Cldn17 in vascular physiology and pathology. Although Cldn17 expression in lung epithelial cells has not been reported, its presence in other epithelial cells [29,30,31,32,33] suggests that it is likely expressed in lung epithelial cells as well. In agreement with this, our initial observations using the Miles assay and Matrigel plug assay established that Cldn17 deficiency compromises vascular integrity, leading to increased dye and hemoglobin extravasation. This increased vascular permeability likely contributes to the pulmonary edema and lung injury observed in the *Cldn17^−/−^* mice. Histological analysis revealed significant alveolar flooding, inflammation, edema, and tissue damage, suggesting that Cldn17 plays a crucial role in maintaining the endothelial barrier function, and its absence predisposes the lungs to injury.

As such, the *Cldn17^−/−^* mice exhibited no significant phenotypic abnormalities, except for being noticeably smaller at birth [13]. However, by 8–12 weeks of age, their body sizes and weights reached parity with the wild-type mice, and no developmental abnormalities were observed [13]. The *Cldn17^−/−^* mice exhibited kidney abnormalities and ionic imbalances in the blood, which is consistent with the expected effects of its paracellular anion channel properties. Beyond these findings, the mice and their breeding capabilities were comparable to the wild-type mice [13]. However, the *Cldn17^−/−^* mouse kidneys revealed acute kidney injury (AKI) without significant kidney edema [13], a finding further confirmed in the current study, as no Evan’s blue dye extravasation was observed in the kidneys as compared with the lungs and skin. This suggests that Cldn17 deficiency may contribute to lung injury through mechanisms independent of vascular permeability. Notably, the kidneys and lungs exhibit significant crosstalk due to shared physiological and molecular pathways [34]. While the precise mechanisms underlying this interaction remain unclear, dysfunction in either organ can exacerbate disease progression [35]. AKI presents a substantial clinical challenge, particularly in the context of ALI, where both experimental and clinical studies have demonstrated a strong association between kidney–lung interactions and poor survival outcomes [36]. Clinically, ALI and its associated interventions to stabilize gas exchange often disrupt kidney homeostasis [37]. Beyond systemic immune activation and inflammatory mediator release, ALI induces oxygen imbalance, impairing renal autoregulation [34]. Hypoxia in the renal system, compounded by ALI-induced hypercapnia, leads to renal vasoconstriction and activation of the renin-angiotensin–aldosterone system [34]. Conversely, AKI profoundly affects pulmonary physiology by disrupting fluid balance, acid–base equilibrium, and vascular tone [37]. The accumulation of inflammatory cytokines due to impaired renal clearance further exacerbates ALI pathogenesis [38]. This bidirectional organ dysfunction is characterized by excessive vascular permeability, immune cell infiltration, and tissue inflammation, ultimately leading to multi-organ failure [36].

To find reasons directly linked to Cldn17 and lung injury in the *Cldn17^−/−^* mice, we performed RNA sequencing analysis of the *Cldn17^+/+^* and *Cldn17^−/−^* mouse lungs, which revealed valuable insights into the potential molecular mechanisms underlying the observed phenotype. The distinct separation between the *Cldn17^+/+^* and *Cldn17^−/−^* lungs in the PCA plot signified profound transcriptional changes. Differential gene expression analysis revealed significant dysregulation of the genes involved in lipid metabolism and transport, hinting at a potential link between Cldn17 and these pathways. Subsequent GO and pathway enrichment analyses underscored the significant impact of Cldn17 deficiency on immune-related pathways, including leukocyte migration, interferon–gamma response, and antigen processing and presentation. The Reactome pathway analysis further highlighted the involvement of innate and adaptive immune responses, neutrophil degranulation, and IL-17 signaling. These findings strongly suggest that Cldn17 is crucial for immune homeostasis and that its deficiency disrupts the delicate balance of immune regulation. Functional enrichment analysis corroborated these findings, highlighting the enrichment of pathways related to antigen processing, IL-17 signaling, and viral protein interactions with cytokine receptors, further emphasizing the immunological consequences of Cldn17 loss.

Further exacerbation of LPS-induced lung injury in the *Cldn17^−/−^* mice solidified the importance of Cldn17 in protecting against inflammatory insults. In general, ion channels [39] and Cldns [18] are implicated in the modulation of inflammation potentially by maintaining ionic balance across the blood–tissue barriers or by other mechanisms involving extracellular vesicles [19,40,41]. The direct link between Cldn17 deficiency in the mice’s lungs and inflammation is also supported by our observations of increased granulocytes and lymphocytes in the *Cldn17^−/−^* mouse blood compared with the *Cldn17^+/+^* mice, indicating a state of leukocytosis. The blood analysis revealed a reduction in platelet count, further supporting the notion of immune dysregulation and potential thrombocytic involvement. The increased neutrophils, lymphocytes, eosinophils, and basophils provide a more granular view of the immune cell populations affected by Cldn17 deficiency.

Pathological analysis of the *Cldn17^−/−^* mice revealed phenotypic features reminiscent of Vascular Leak Syndrome (VLS), a condition characterized by increased vascular permeability, fluid and protein extravasation, interstitial edema, and organ failure [42]. Hallmark signs of VLS, such as splenomegaly, elevated white blood cells, increased globulin levels, and reduced albumin, were observed in the *Cldn17^−/−^* mice. Additionally, ALI and AKI, common complications of VLS, were also evident in the *Cldn17^−/−^* mice. Previous studies have identified Cldn17 as one of 17 rare copy-number variations associated with isolated posterior urethral valves, a leading cause of bilateral renal obstruction [43]. In summary, the function of Cldn17 extends beyond its role in regulating endothelial and epithelial barrier integrity.

While this study provides key insights into the role of Cldn17 in vascular integrity and immune regulation, several limitations remain. The precise mechanisms driving increased vascular permeability in the *Cldn17^−/−^* mice require further investigation to distinguish direct endothelial dysfunction from secondary inflammatory effects. Bulk RNA sequencing lacks cell-type resolution, and future studies using single-cell approaches could provide more precise insights. Given the kidney–lung crosstalk, potential renal contributions to lung injury warrant further exploration. Finally, while features of VLS were observed, additional imaging and functional assays are needed to confirm endothelial ultrastructural changes. Although this study provides strong evidence for the role of Cldn17 in vascular integrity and immune regulation, further investigation is warranted. Future studies should focus on elucidating the precise molecular mechanisms by which Cldn17 exerts its protective effects. Investigating the interaction between Cldn17 and other tight-junction proteins, as well as exploring the signaling pathways involved in Cldn17-mediated immune modulation, would be valuable. Furthermore, examining the potential therapeutic implications of these findings, such as targeting Cldn17 or its downstream effectors, could pave the way for novel treatments for conditions characterized by vascular leak and immune dysregulation. Finally, the association of Cldn17 with posterior urethral valves warrants further investigation to understand the connection between these seemingly disparate phenotypes.

## 4. Materials and Methods

### 4.1. Animals

The Charlie Norwood Veterans Affairs Medical Center Institutional Animal Care and Use Committee (protocol # 19-04-114 and 20-01-118) approved all mouse experiments. These studies adhered to ARRIVE guidelines for reporting animal research. Anesthesia was induced via the intraperitoneal injection of ketamine and xylazine, and euthanasia was performed using CO_2_ asphyxiation followed by cervical dislocation. To generate knockout mice [13] (Envigo, Indianapolis, IN, USA), 10 sgRNAs were designed for CRISPR/Cas9-based gene editing in C57BL/6 zygotes. The most effective sgRNA, selected for minimal off-target effects, was incorporated into a ribonucleoprotein complex with Cas9 endonuclease. This complex was introduced into C57BL/6 zygotes, which were then implanted into pseudo-pregnant females. The resulting progeny were screened for the intended mutation using genomic PCR and DNA sequencing. Up to two transgenic founders were selected and bred with C57BL/6 mice to generate F1 heterozygous (*Cldn17^+/−^*) offspring, confirmed through PCR-based genotyping and sequencing. On-target and off-target analyses, including sequencing of the top 10 predicted off-target sites, were performed in F1 animals. Finally, the *Cldn17^+/−^* mice were bred to obtain homozygous *Cldn17^−/−^* mice for the study.

### 4.2. Cell Culture and Reagents

Immortalized human microvascular lung endothelial (HMLE) cells were cultured in a complete human endothelial cell medium (Cat# H1168, Cell Biologics, Chicago, IL, USA). Cells between passages around 14–21 were utilized for the experiments. Plastic culture wares, chemicals, and all other reagents used in the study were obtained from Fisher Scientific, Hampton, NH, USA. VEGF (Cat # BT-VEGF) and angiopietin-1 (Cat # 923-AN-025/CF) were purchased from R&D Systems, Minneapolis, MN, USA at 20 ng/mL doses for 1, 12, and 24 h.

### 4.3. Western Blot Analysis

The cellular monolayers were scratched and lysed using a mixture of protease and phosphatase inhibitors in RIPA buffer (Roche, Basel, Switzerland). The protein concentration was determined using a DC protein assay (Bio-Rad, Hercules, CA, USA), and approximately 30–40 μg of proteins in Laemmli buffer were used for the experiments. Western blotting was performed as previously described [25,44,45]. The antibodies used and their respective dilution ratios were as follows: Anti-Cldn17 (1:1000, Cat No. LS-C30623, LSBio, Newark, CA, USA), Anti-Cldn4 (1:1000, Cat No. EPRR17575, Abcam, Waltham, MA, USA), Anti-Cldn9 (1:1000, Cat No. ab192398, Abcam), and Anti-Cldn5 (1:1000, Cat No. 4C3C2, Thermo Scientific, Waltham, MA, USA). The monoclonal anti-β-actin antibody (1:10000, Cat No. 5174) was purchased from Sigma, St. Louis, MO, USA. The secondary antibodies, including anti-mouse (Cat No. 170-6516) and anti-rabbit (Cat No. 170-6515), were procured from Bio-Rad (Hercules, CA, USA). Band intensity quantifications were performed using the National Institutes of Health (NIH) ImageJ software (Version 1.53).

### 4.4. Miles Assay

Vascular permeability in vivo was assessed using the Miles assay as previously described [11,46,47]. The *Cldn17^+/+^* and *Cldn17^−/−^* animals were injected with 50 µL of 1% Evan’s blue dye intravenously, followed by a 30-min or 3-h interval, respectively, before euthanasia with CO_2_. The animals were then perfused with 1X PBS to remove excess dye, and organs (lung, kidney, and ear) were collected and stored in formamide at 42 °C for 24 h. The absorbance of the formamide was measured at 610 nm by spectrophotometry, with values normalized to the organ weight to determine vascular leakage.

### 4.5. Matrigel Assay

The Matrigel plug assay was performed following a procedure outlined in our previous study [47]. On day 7, the animals were euthanized, and the Matrigel plugs were collected. The plugs were then digested using 5 mL of Drabkin’s reagent (Sigma D5941), and neo-vessel formation was quantified using a hemoglobin assay according to the manufacturer’s protocol.

### 4.6. Analysis of Lung Injury and Pulmonary Edema

Age- and gender-matched WT and *Cldn17^−/−^* mice were treated with LPS (10 mg/kg) for 24 h (i.p.) and euthanized to collect lungs for histological analysis and assessment of pulmonary edema [48]. The right superior lobe of the lung was fixed in 4% paraformaldehyde, and ten-μm thick sections were prepared for H&E staining. Histological evaluation of these slides was conducted by blinded reviewers, who scored tissue fields on a scale of 1 to 5, with 1 indicating minimal injury and 5 representing the most severe injury. The average scores were used for statistical analysis. Interstitial congestion, inflammatory cell infiltration, and consolidation were considered in the lung injury scoring. The left lung lobe was weighed fresh and then incubated for 72 h at 80 °C on sterile paper to remove moisture. The dry weights were recorded, and the wet/dry ratios were calculated to assess edema or fluid content.

### 4.7. Flow Cytometry Analysis

Blood was collected using the retro-orbital technique into EDTA-treated collection tubes. Approximately 100 µL of blood was mixed with 1 mL of ACK lysing buffer and incubated at room temperature for 3 to 5 min to lyse the red blood cells. After centrifugation at 300× *g* for 5 min at room temperature, the white blood cells were collected as a pellet, and the supernatant was aspirated. The pellet was washed with 0.5 mL ice-cold PBS and centrifuged again at 300× *g* for 5 min at 2–8 °C. The cells were then resuspended in PBS for flow cytometry. Forward vs. side scatter plotting was performed on these samples to achieve a cell count of 10,000 using the Cytoflex LX from Beckman Coulter (Indianapolis, IA, USA) [49].

### 4.8. RNA-Seq and Bioinformatic Analysis

Lungs were isolated from the WT and *Cldn17^−/−^* animals and stored in Trizol^®^ before freeze-drying. RNA isolation was performed on the samples, and total RNA purity and concentration were assessed using spectrophotometry with the NanoDrop ND-1000 (ThermoFisher). RNA quality was evaluated using the Agilent 2100 Bioanalyzer (Agilent Technologies, Santa Clara, CA, USA), ensuring an RNA Integrity Number (RIN) greater than 5. Total RNA was processed for cDNA library preparation using the TruSeq Stranded Total RNA kit (Illumina, San Diego, CA, USA), which depletes cytoplasmic ribosomal RNA. Briefly, 800 ng of total RNA was treated with rRNA Removal Mix to deplete the rRNA. After purification, the RNA was fragmented into 200–300 bp segments and converted into cDNA fragments. A single ‘A’ base was added to the cDNA fragments, followed by ligation of the adapter. The products were purified and enriched through PCR to create the final cDNA library. The library was assessed for quality with the Bioanalyzer and quantified using Qubit (ThermoFisher). The libraries were pooled and sequenced on the NextSeq500 sequencing system using a 75-cycle paired-end protocol. BCL files generated by the NextSeq500 were converted to FASTQ files for downstream analysis. Reads that passed quality control were aligned to the reference genome using the STAR aligner. The resulting BAM files were imported into Cufflinks and Cuffdiff tools from the Tuxedo Suite for differential gene expression analysis, with log2 fold changes and q-values calculated for replicates. Genes with *p*-values <0.05 were considered for further bioinformatics analysis.

Analysis of gene distribution, such as principal component analysis (PCA), volcano plot, and heatmap analysis, was performed using R version 4.3.2 and SRplot. Detailed pathway analyses were performed on genes that were differentially expressed in the lungs of *Cldn17^−/−^* compared to WT mice employing the STRING database [50] and SRplot [51]. The pathway analysis of these genes was conducted on multiple databases such as the Kyoto Encyclopedia of Genes and Genomes (KEGG), Wiki, and Reactome. The gene set enrichment analysis (GSEA) and GO databases were used to scrutinize the cellular components, biological processes, and molecular functions of identified proteins and signaling pathways. The STRING database was utilized to demonstrate the gene-gene interactions [50]. Tissue-wide expression data were collected from the GTEx portal (https://www.gtexportal.org/home/ (accessed on 8 March 2025)).

### 4.9. Statistical Analysis

All the data are presented as mean ± SD or mean ± SEM. The ‘n’ value for each figure implies the number of samples in each group, which was determined by power analysis. All the data were analyzed by parametric testing using the student’s unpaired *t*-test or one-way ANOVA using the GraphPad Prism 6.01 software. The Chi-square test was also used wherever applicable. Data with *p* < 0.05 were considered significant.

## Data Availability

All the data have been included in the manuscript, and the raw data are included in the Supplemental File.

## Supplementary Materials

The following supporting information can be downloaded at https://www.mdpi.com/article/10.3390/ijms26083612/s1.
