# Supplementary material for: Claudin-17 Deficiency Drives Vascular Permeability and Inflammation Causing Lung Injury
Source: Int J Mol Sci. 2025 Apr 11;26(8):3612. doi: 10.3390/ijms26083612 (PMC12027279; doi:10.3390/ijms26083612)
Supplement: Supplementary file 1 [file ijms-26-03612-s001.zip › ijms-3509211-supplementary.pdf]

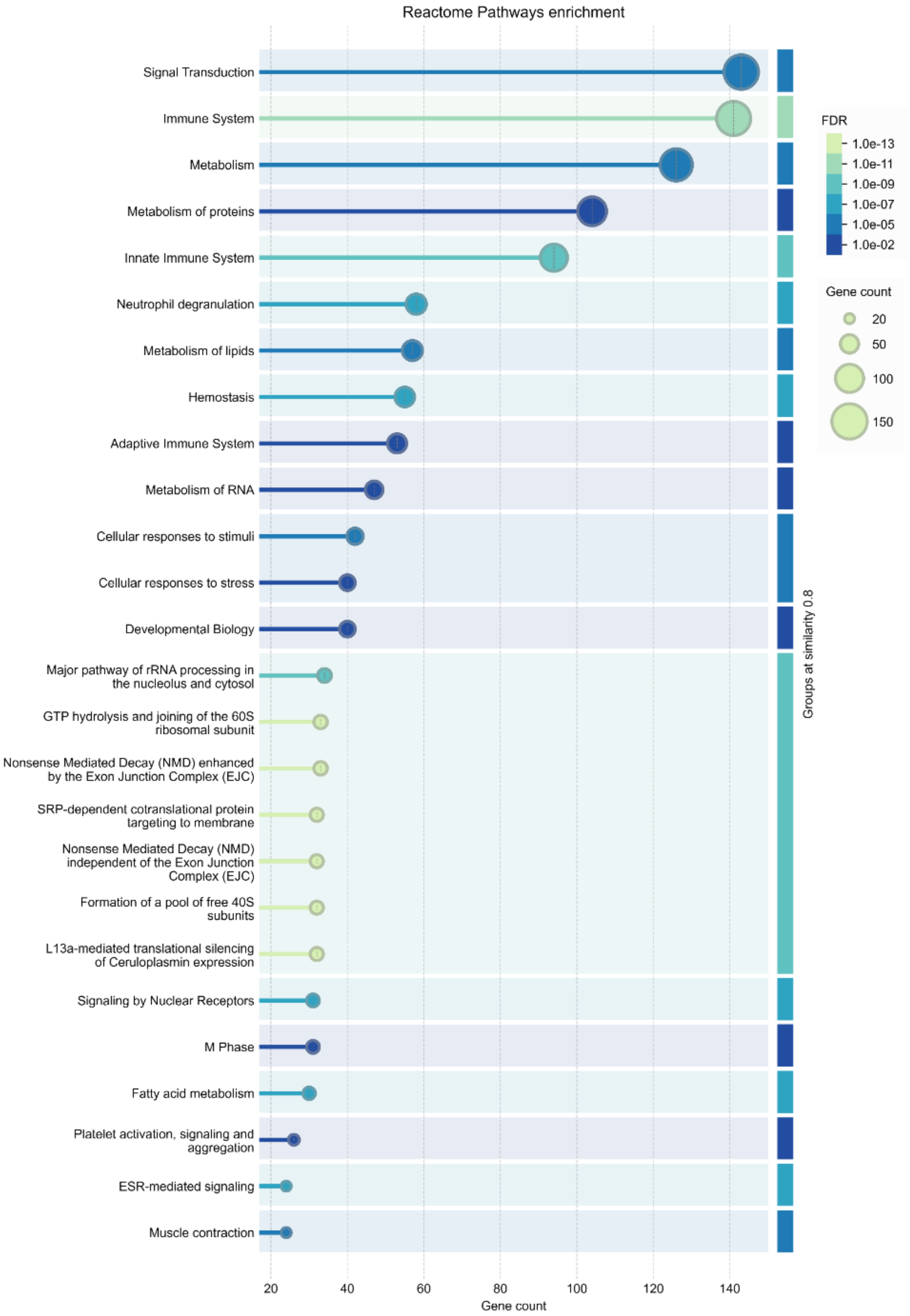

Supplemental Figure S1. Reactome pathway enrichment analysis showing key pathways affected by Cldn17 deficiency. The size of the circles represents the number of genes involved, while the color intensity corresponds to the false discovery rate (FDR).

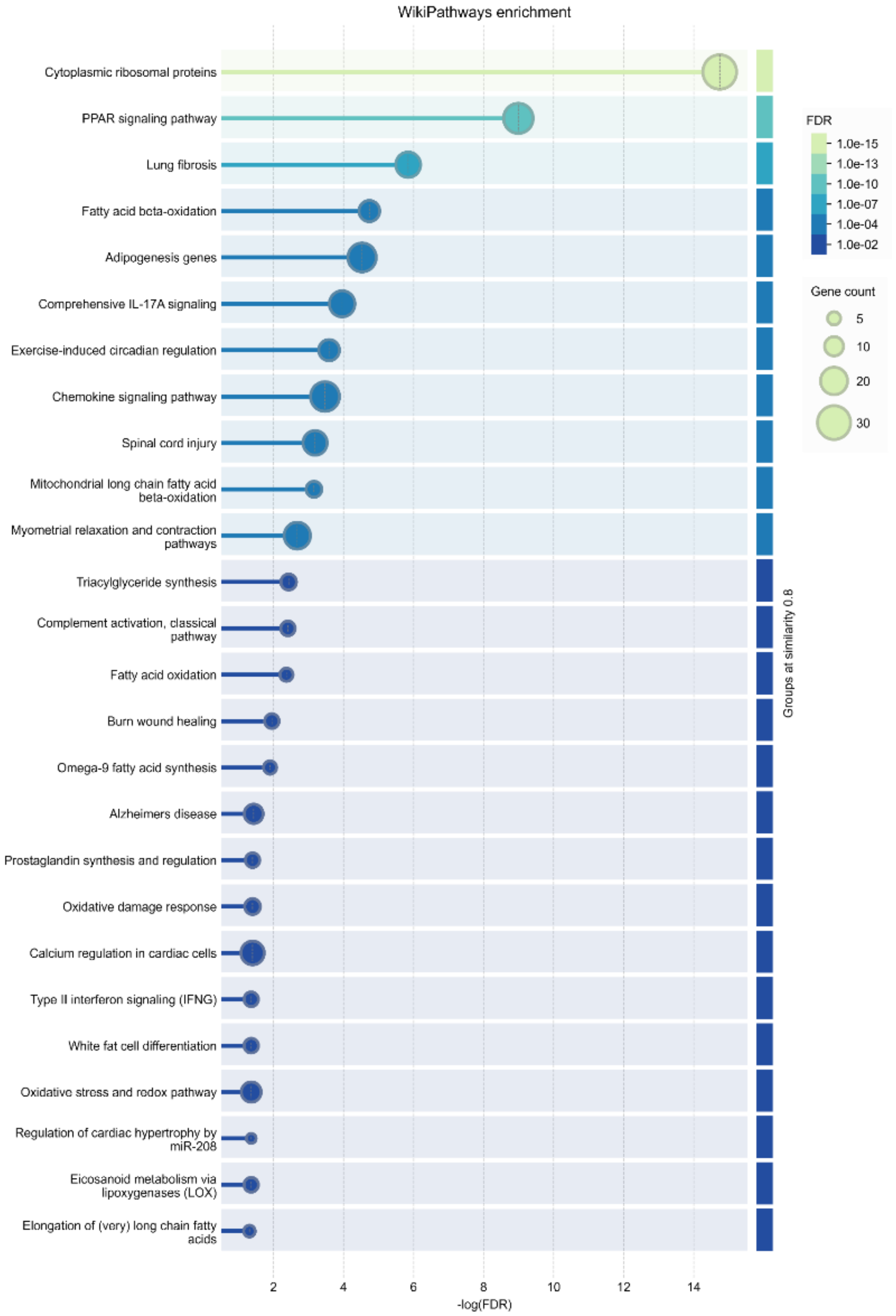

Supplemental Figure S2. Wiki pathway enrichment analysis showing key pathways affected by Cldn17 deficiency. The size of the circles represents the number of genes involved, while the color intensity corresponds to the false discovery rate (FDR).

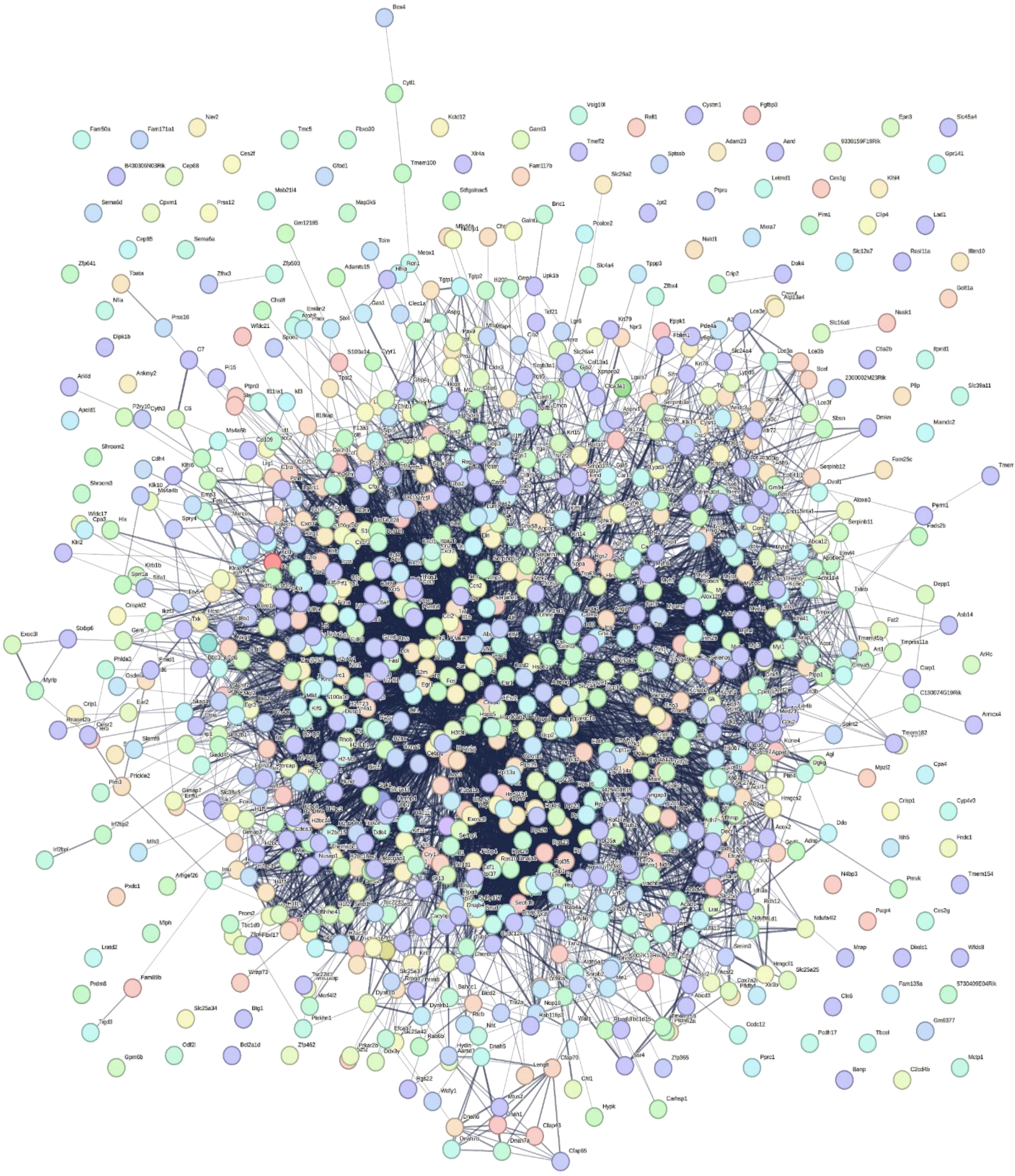

Supplemental Figure S3. STRING analysis showing molecular interactions in DM lungs
